# Stain Style Transfer for Histological Images Using S3CGAN

**DOI:** 10.3390/s22031044

**Published:** 2022-01-28

**Authors:** Jiann-Shu Lee, Yao-Xian Ma

**Affiliations:** Department of Computer Science and Information Engineering, National University of Tainan, Tainan 700, Taiwan; polo19961202@gmail.com

**Keywords:** CycleGAN, stain transfer, WSI

## Abstract

This study proposes a new CycleGAN-based stain transfer model, called S3CGAN, equipped with a specialized color classifier structure. The specialized color classifier can assist the generative network to conquer the existing challenge in GANs, namely the instability of the network caused by the insufficient representativeness of the training data in the initial stage of network training. The color classifier is pretrained, hence it can provide correct color information feedback to the generator during the initial network training phase. The augmented information from color classification enables the generator to generate superior results. Owing to the CycleGAN architecture, the proposed model does not require representative paired inputs. The proposed model uses U-Net and a Markovian discriminator to enhance the structural retention ability to generate images with high fidelity.

## 1. Introduction

Medical imaging techniques, such as ultrasound imaging, computed tomographic scanning, positron emission tomography, single-photon emission computed tomography, magnetic resonance imaging, and thermal imaging, are used for the early detection of cancer. However, histopathological biopsies are currently the only recognized method for cancer diagnosis. Biopsy technologies can now scan the entire tissues on microscope slides and produce whole slide images (WSIs). These images can then be used for automated image analysis. The production of WSIs often results in varying stain colors due to reasons such as using different batches of staining reagents, different suppliers of staining reagents, samples with different thicknesses, different staining conditions, and different image scanners. Variations in color diminish the performance of automated image analysis.

Conventional image processing techniques [1,2,3,4] have been used to resolve the problem of stain variations; however, they produce unfavorable results due to the limitations of the image processing methods. These limitations come from improper color mapping, failure to take spatial features into account, or the need to choose a representative reference image. Recently, generative adversarial networks (GANs) [5] exhibit amazing results in generating high-fidelity images. GANs consist of two networks, a generator and a discriminator. They both play an adversarial game during the training stage, where the generator tries to cheat the discriminator by generating data similar to those in the training set. The discriminator tries not to be cheated by distinguishing between real and fake data. In the testing stage, the trained generator is used to generate data that is highly realistic to real data. Conditional GANs (CGANs) [6] are a natural extension of GANs in which the limitation factors can be set for the input of the network, forming paired inputs, to further control the generated data. This controllable mechanism is suitable for generating specific images. However, one inherent weakness of CGAN is that the input data must be paired. In [7], a CGAN-based approach was proposed to solve the stain variation problem, but their stain transfer quality was not ideal. This is because they transfer stain style by colorizing gray images; however, even the same tissue can produce different grayscale values under different staining conditions. In recent years, CycleGAN [8] was developed to transfer the characteristics of one image to another, called style transfer, via treating this transfer task as an image reconstruction problem. This approach naturally solves the problem that CGAN is limited by the need for paired inputs. In [9], a CycleGAN-based approach was proposed to normalize the stain variation. However, the fidelity of the transferred images was insufficient and sometimes produced abnormal tones. These problems arose from two insufficiencies of the generator, one was the structural retention ability, the other was the color–texture coherence.

An excellent pathological image staining conversion does not change the texture structure of the original image, but the new staining must be able to color according to the texture of the original image. The former is reflected in the structural retention ability, while the latter is reflected in the color–texture coherence for a generator. Based on this consideration, we propose a new neural network model called S3CGAN (specialized-color-classifier CycleGAN). The CycleGAN architecture is adopted to eliminate the need of selecting representative paired inputs for CGAN-based approaches. The structural retention ability is enhanced through the U-Net [10] and the Markovian discriminator [11]. The U-Net is used to endow the generator with pixel-level description capability and the Markovian discriminator provides distributed locality constraints on pathological image structures. A specialized color classifier is utilized to offer coherent color–texture information to the generator. The proposed model produces superior stain transfer outcomes and effectively resolves the color variation problem for pathological images.

## 2. Related Work

### 2.1. Conventional Color Normalization

To overcome the problem of stain variation, Reinhard et al. [1] proposed a color correction method in which the colors of one image were applied to another. In this method, the stain ratio between the source image and the target image is assumed to be similar. The source image is converted from the RGB color space to lαβ color space. The color histograms of the source and target images are then aligned to complete the stain transfer. Spatial information is not considered in this method; therefore, incorrect stain transfers often occur if the source and target images have different stain ratios. Macenko et al. [2] improved the method of Reinhard et al. [1] and conducted stain separation for stain normalization. First, the source image is converted from the RGB color space to the optical density space. Next, color deconvolution is performed to calculate the stain matrix, which indicates how each stain contributes to the RGB colors. Finally, the stain matrix is used for stain normalization. Vahadane et al. [3] used a structure-preserving color normalization algorithm, which is a spectral matching method, for color correction. This method is only suitable for hematoxylin and eosin (H&E) stains because the colors are rendered differently in other stains. Khan et al. [4] used color classifiers to calculate the contributions of different stains to the RGB colors and then used stain matrices for stain normalization. Although this method achieved a superior performance to that of Reinhard et al. [1], it has certain limitations because it requires a reference image for analysis.

### 2.2. Color Normalization Methods Based on GANs

**Figure 1:**
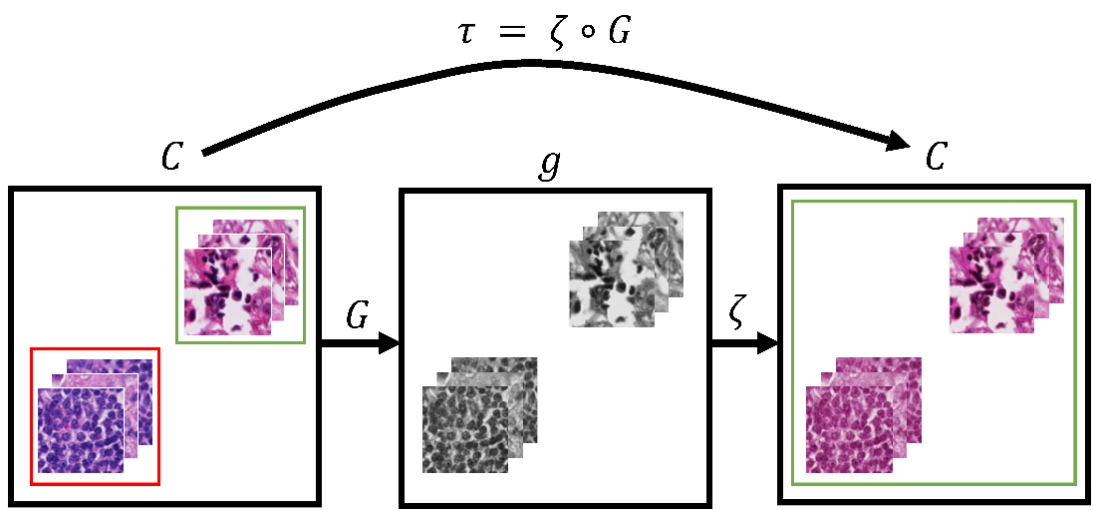
Overview of the stain style transfer network proposed by Cho.

Cho et al. proposed a stain transfer network based on a CGAN (Figure 1) [7]. The network τ is composed of two transformations: gray-normalization G and style-generator ζ. G standardizes each stain style of color images from different institutes and ζ colorizes gray images following the stain style of certain institutes. This method does not require additional corresponding labels for support; however, the stain transfer outcomes of this method are not ideal because even the same tissue can produce different grayscale values under different staining conditions.

**Figure 2:**
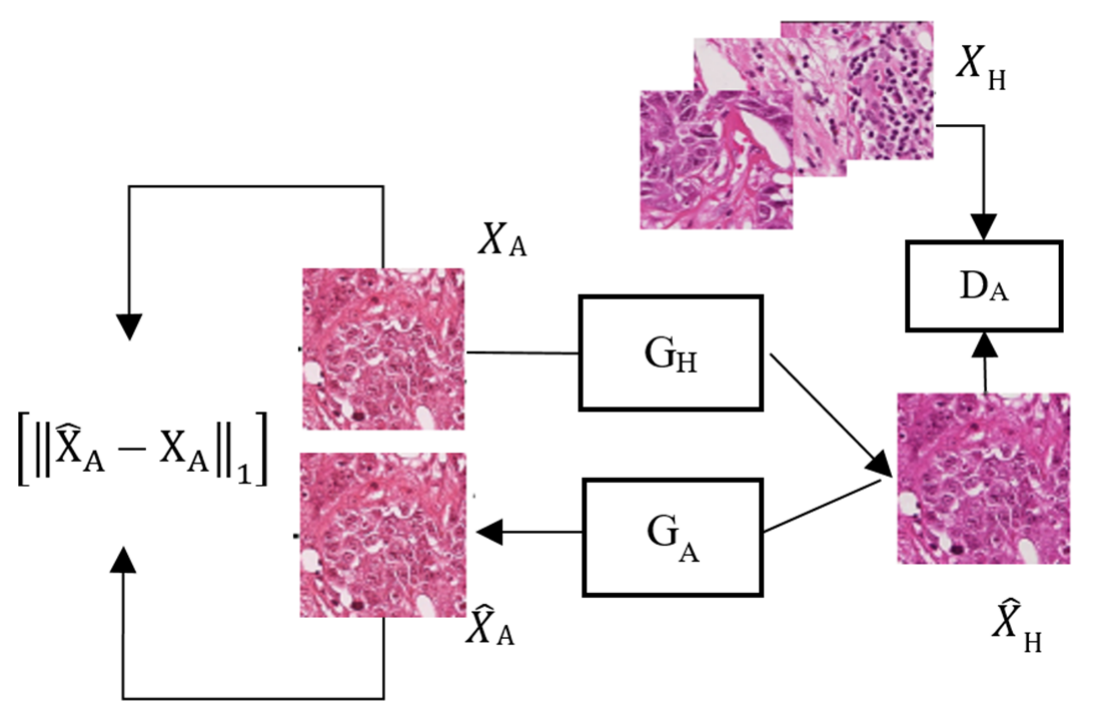
System architecture of StainGAN.

Shaban et al. [9] proposed a new stain transfer network called StainGAN (Figure 2), which uses the original CycleGAN architecture for modeling. This network does not require paired data and additional task labels. However, the image fidelity after the stain style conversion is not ideal, and sometimes local color drifts appear. Preserving high fidelity after stain style conversion for pathological images is crucial for disease diagnosis. Distortion of the pathological image during stain transfer increases the risk of misdiagnosis. In addition, the network does not optimize the stability of its training process; thus, its training process often fails.

## 3. Proposed Method

### 3.1. System Architecture

**Figure 3:**
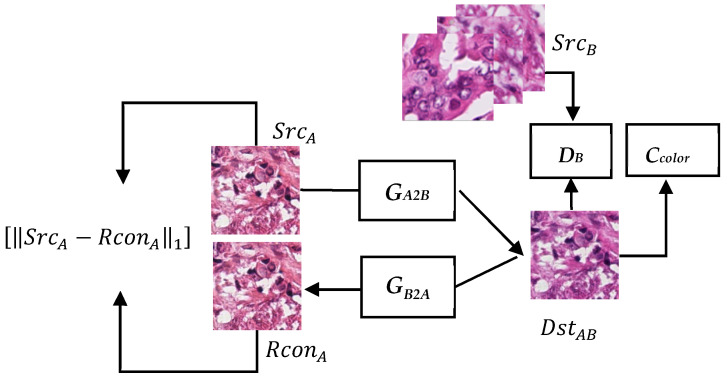
System architecture of the S3CGAN.

To overcome the aforementioned problems, we propose a new stain transfer model called the S3CGAN. The input of this model comprises RGB channels so that it can avoid the problems associated with the method presented in [7], in which only brightness information is used. The proposed model uses U-Net, which has excellent structural expression ability, as the generative network architecture. The Markovian discriminator [11], which uses local receptive fields, is used to preserve the tissue structure in the images. In addition, we propose a specialized color classifier to assist the generative network to generate images with color characteristics similar to the target domain and to stabilize the network in the initial training phase. WGAN–GP (Wasserstein GAN with gradient penalty) [12] is used to further enhance the stability of network training. The architecture of the proposed S3CGAN is shown in Figure 3, where the scenario is based on the conversion of type A stain to type B stain and vice versa. The operation process of this architecture is briefly described as follows, and the more detailed operation details will be explained in the following sections. The source image with type A stain, denoted as ***Src_A_***, is converted to a new style with type B stain, denoted as ***Dst_AB_***, via the generator ***G_A2B_***. The converted result Dst_AB_ is forward to three different parts, namely ***G_B2A_***, ***D_B_***, and ***C_color_***. ***G_B2A_*** is another generator used to converts ***Dst_AB_*** back to the original type A stain, denoted as ***Rcon_BA_***. ***D_B_*** is a discriminator used to check if ***Dst_AB_*** looks like type B stain. ***C_color_*** is a pretrained color classifier used to assess the likelihood of Dst_AB_ being type B stain from the color perspective. During the training stage, ***G_A2B_***, ***G_B2A_***, and ***D_B_*** are updated at the same time. For ***D_B_***, its update driving force comes from the loss of distinguishing between real image (***Src_B_***) and fake image (***Dst_AB_***). For ***G_A2B_***, its update driving force comes from three losses, one from the reconstruction quality (***Src_A_*** vs. ***Rcon_BA_***), one from the success rate of ***G_A2B_*** cheating ***D_B_***, and the other from the color possible correctness of ***Dst_AB_***. ***G_B2A_*** and ***D_A_*** undergo the same process in the reverse direction. Detailed explanations of the different parts of the proposed network are provided in the following sections.

### 3.2. Generator

**Figure 4:**
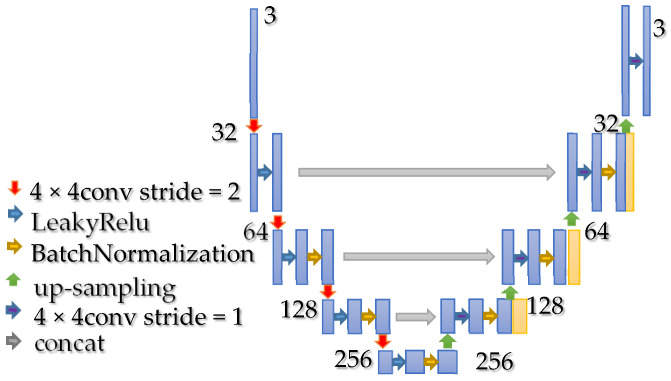
Architecture of U-Net.

In this study, the S3CGAN comprises two independent generators, one of which is responsible for transferring the source domain image to the target domain and the other of which is responsible for transferring the target domain image to the source domain. In contrast to the method used in [9], in which ResNet was used as the main generative architecture, we used U-Net [10] because it has excellent image detail expression ability. This attribute is crucial because if tissue sample images are distorted during the stain transfer, the accuracy of disease diagnosis may decrease. The architecture of the U-Net is displayed in Figure 4.

### 3.3. Discriminator

**Figure 5:**
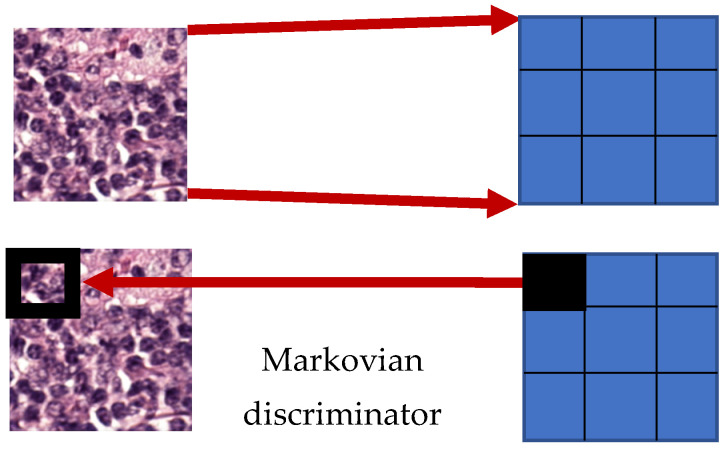
Markovian discriminator.

The discriminators used by conventional GANs output a single value, which can only reflect the overall quality of the generated image but cannot reflect the quality of different parts. That is, the receptive field of conventional GANs is the entire image, which often results in low image quality in certain parts of the generated image. This property is not appropriate for histological imaging, which requires the presentation of details and high discrimination. The Markovian discriminator outputs a group of values, each of which represents the image quality of a part of the generated image (Figure 5). This discriminator maps the input onto an N × N-sized matrix. Each value in the matrix represents the similarity between a patch and the corresponding target image feature, which can be used to optimize each patch. This study used the Markovian discriminator to enhance the structural retention quality after stain transfer.

### 3.4. Specialized Color Classifier

GAN models mostly exhibit a poor performance if training samples that lack representativeness are used during their initial training phase. To produce high-quality stain images via using generators, the texture and color distributions must be known. In conventional GAN architectures, the distribution information is indirectly provided by the discriminator in its feedback. Training generators to learn both the texture and color distributions is difficult if the discriminator only provides two feedback categories: real and fake. A method to resolve this problem is to increase the amount of information in the feedback. The information can be enhanced via using a color classifier to provide color classification results for a generated image. Such a classifier can be constructed using two approaches. The first one involves embedding color classification functions into the discriminator. This method, also known as embedded classification, is used in the auxiliary classifier GAN (AC-GAN) [13]. The second one is used in this study, which involves developing a specialized color classifier. In the first approach, the discriminator must be optimized for discrimination and classification tasks during training, which is more challenging. In the second approach, a pretrained color classifier ***C_color_*** is used to feedback color information to the generator. Because the color classifier has been trained, it can provide correct color information feedback to the generator during the initial network training phase, which is the most crucial phase during network optimization and the phase in which network instability is most likely to occur. The color classifier can assist the optimization process of the generator. This classifier plays a similar role as a “color consultant.” It can also reduce the burden of the discriminator, facilitate the optimization of discrimination tasks, and stabilize the network training process.

**Figure 6:**
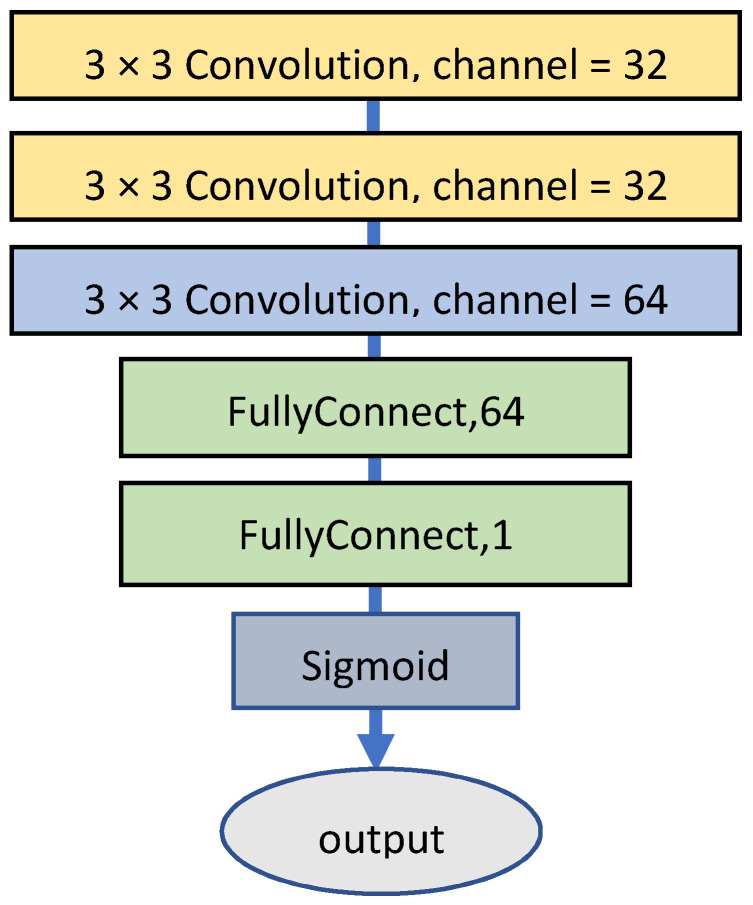
Architecture of the specialized color classifier.

***C_color_*** is a simple binary classification network with a lightweight architecture, as shown in Figure 6. ***C_color_*** uses images from the source and target domains as training data. After training, ***C_color_*** can determine if an input image belongs to the source or target domain. The augmented information of color classification enables the generator to generate superior results during the initial training phase. Even if the training samples with less stain representativeness are used for the discriminator during the initial training phase, owing to the augmented color classification information the bias coming from the training samples can still be corrected.

### 3.5. Training Process

Step 1: A total of 3000 patches each were selected from type A and type B stain images. These patches were used as training samples to train the color classifier network ***C_color_***.

Step 2: This step corresponds with the training phase of the CycleGAN. We used the 6000 patches selected from type A and type B stain images as training data. The training data set is different from that of the first step. Figure 3 corresponds to the use of the ***Src_A_*** type A stain images as the input. We first input the ***Src_A_*** images by batches into the generator ***G_A2B_*** to produce a group of fake type B stain images (***Dst_AB_*** images). The ***Dst_AB_*** images were then input into the generator ***G_B2A_*** to reconstruct the original input image ***Rcon_A_***. Subsequently, the generated ***Dst_AB_*** images and original real type B stain images (***Src_B_*** images) were used as training data to train the discriminator ***D_B_*** such that ***D_B_*** could learn to recognize whether the input images were real type B stain images. The WGAN-GP loss function *L_advD_* used for training is defined in (1), where $P_{\hat{A}}$ is the distribution calculated from the linear combination of the real distribution $P_{\left(real\left(A\right)\right)}$ and generative distribution $P_{\left(gen\left(\tilde{A}\right)\right)}$. The gradient penalty in this loss can boost the stability during network training.

Step 3: This step corresponds to the second part of the training phase of the CycleGAN. The generator was trained after training the discriminator in step 2. We used three loss functions, namely *L_adv_*, *L_cyc_*, and *L_color_*, to optimize the generator during generator training. The generated ***Dst_AB_*** images were input into the discriminator ***D_B_*** to obtain the adversarial loss *L_adv_*, reflecting the difference between the generated and the real type B stain images. The definition of the loss function *L_adv_* is shown in Equation (2). The restored image ***Rcon_A_*** and the original ***Src_A_*** images were used to calculate the mean absolute error, which was used to quantify the cyclic loss *L_cyc_*. In addition, the generated ***Dst_AB_*** images were input into ***C_color_*** to calculate the cross entropy, denoted as *L_color_*, between the classification results and the correct outcomes to reflect their similarity. Equations (1)–(5) present expressions for the loss functions and target loss functions used in the network training.

The generator ***G_B2A_*** and discriminator ***D_A_*** were trained using the same way but in the reverse direction. The training stopped after multiple training iterations when the generated image became stable.

(1) $${ℒ}_{advD}=E_{\tilde{A}\sim P_{gen\left(\tilde{A}\right)}}\left[D_{A}\left(\tilde{A}\right)\right]-E_{A\sim P_{real\left(A\right)}}\left[D_{A}\left(A\right)\right]+10E_{\hat{A}\sim P_{\hat{A}}}\left[{\left(||\nabla_{\hat{A}}D_{A}{\left(\hat{A})|\right|}_{2}-1\right)}^{2}\right]$$

(2) $${ℒ}_{adv}=E_{A\sim P_{data}\left(A\right)}\left[log^{\left(1-D_{B}\left(G_{A2B}\left(A\right)\right)\right)}\right]$$

(3) $${ℒ}_{cyc}\left(G_{A2B},G_{B2A}\right)=E_{A\sim P_{data}\left(A\right)}\left[{\left|\left|G_{B2A}\;\left(G_{A2B}\;\left(A\right)\right)-A\right|\right|}_{1}\right]$$

(4) $${ℒ}_{color}=E_{A\sim P_{data\left(A\right)}}\left[log^{\left(C_{color}(G_{A2B}\left(A\right))\right)}\right]$$

(5) $${ℒ}_{obj}={ℒ}_{Adv}+\alpha {ℒ}_{cyc}+\beta {ℒ}_{color}$$

## 4. Experiments

Two datasets were used in this study to evaluate the performance of the proposed model. The Camelyon 16 dataset [14] was used to evaluate the tumor classification performance, and the Mitos-Atypia-14 dataset [15] was used to evaluate the generated image quality and structure fidelity.

### 4.1. Datasets

**Figure 7:**
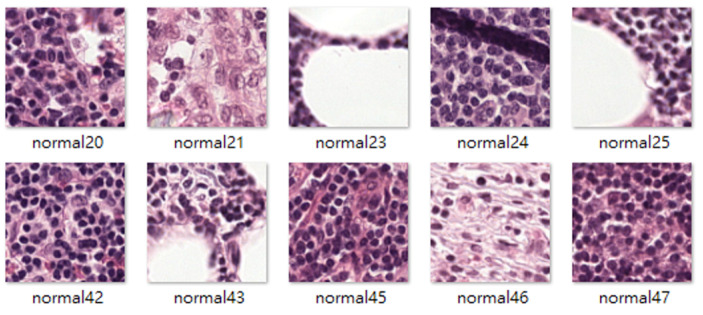
Samples of type B stain images.

**Figure 8:**
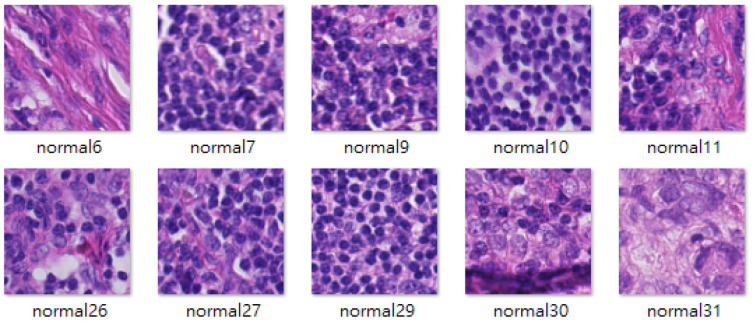
Samples of type A stain images.

The Camelyon16 challenge was held by the International Symposium on Biomedical Imaging, and the goal of this challenge was to detect metastases of breast cancer automatically in H&E-stained lymph node sections. The Camelyon16 dataset includes many WSIs of sentinel lymph nodes that were provided by Radboud University Medical Center (marked as lab 1) and University Medical Center Utrecht (marked as lab 2). This study used 3000 patch images, each from lab 1 and lab 2, to train the stain transfer model. The size of the patch images is 256 × 256 pixels. A basic tumor classifier was used as a test platform to evaluate how stain transfer affected the tumor classification performance. This tumor classifier was trained using the dataset collected by Shaban et al. [9], which contains 14,704 tumor patch images and 14,704 nontumor patch images. The aforementioned training data were labeled type B stain images (Figure 7). The test data comprised 10,816 patch images and were labeled type A stain images (Figure 8).

**Figure 9:**
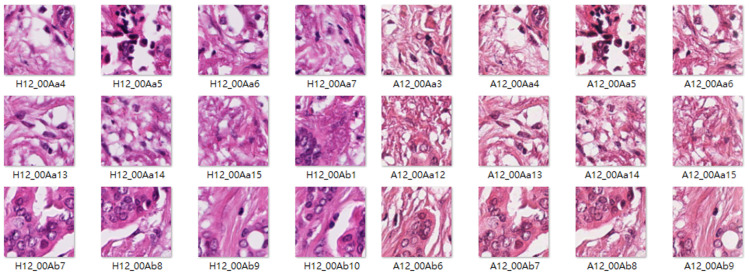
Example images from the Mitos-Atypia-14 dataset.

The Mitos-Atypia-14 dataset comprises 280 H&E-stained biopsy WSIs of the same samples captured using two scanners, namely the Aperio Scanscope XT and Hamamatsu Nanozoomer 2.0-HT, at a magnification of 20× (Figure 9). The training data comprised 3000 patch images each for both scanners. The size of these patch images is 256 × 256 pixels. The test data comprised 4741 images, each scanned by the Aperio Scanscope XT and Hamamatsu Nanozoomer 2.0-HT as the ground truth.

### 4.2. Evaluating the Tumor Classification Performance

This experiment was conducted with the Camelyon16 dataset, and the tumor classification performance obtained for with/without stain style transfer was employed to indirectly verify the effectiveness of the proposed stain transfer method. A simple classification network architecture, the same as Figure 6, was used in this experiment because of its weak generalization capability, such that an excellent classification performance can be obtained only when the input image features are similar to the trained image features. Thus, the classification performance can indicate the similarity between the style-transferred images and the target images. We used the type B stain images [9] to train the tumor classifier. Next, the type A stain images [9] were converted to type B stain images via different stain transfer methods. These transferred type B stain images were input into the trained tumor classifier to evaluate the classification accuracy and indirectly verify the effectiveness of the different stain transfer methods.

**Figure 10:**
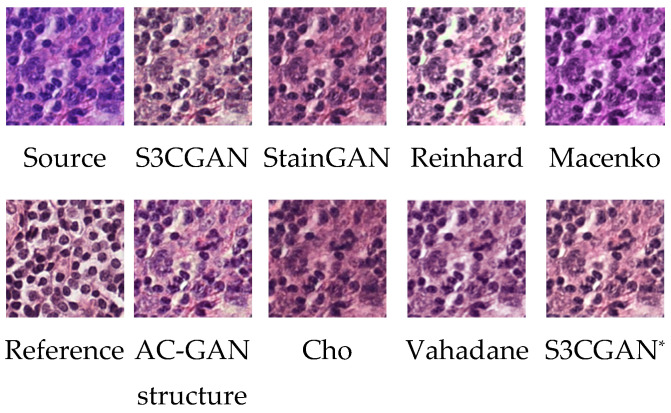
Transfer results obtained with different stain transfer methods.

Figure 10 shows the stain-transferred images using different approaches. We can find that the transferred image using the proposed method has the closest overall tone to the reference image. The results presented in Table 1, annotated as “(simple classifier)”, indicate that the proposed method produced superior classification performance to the other methods. This result indirectly indicates that the produced images by the proposed method are the closest to the training dataset’s type B stain image features. In Table 1, S3CGAN* refers to the S3CGAN without a specialized color classifier. The AUC value of the S3CGAN was 2% higher than that of the S3CGAN*, which proves that the specialized color classifier enhanced not only the stability during network training but also the stain transfer performance. The AUC value of the S3CGAN was also 2% higher than that of the AC-GAN structure, which uses an embedded classifier. This result indicates that the specialized color classifier can improve the overall stain transfer performance. The performance of the S3CGAN was 4% higher than that of the StainGAN. In addition to the effects of the specialized color classifier, the usage of the U-Net and the Markovian discriminator also improved the structural retention ability. To be closer to the clinical use situation, a sophisticated tumor classifier proposed by Jiang et al. [16] was also used. The results are presented in Table 1, annotated as “(complicated classifier)”. As expected, due to the better generalization ability of this classifier, the effect of the differences in the efficacy of the stain conversion methods is relatively limited. Even so, the method proposed in this study still outperforms other methods.

### 4.3. Mitos-Atypia-14 Experiment

The Mitos-Atypia-14 dataset contains biopsy images obtained using two scanners; thus, this dataset can be used for obtaining the ground truth and objectively evaluating the image quality of stain-transferred images. We used the structural similarity index measure (SSIM) [17] to evaluate the similarity between a stain-transferred image and the ground truth. Moreover, we used the PSNR to evaluate the image quality of the stain-transferred images. We converted the format of the images scanned by the Aperio Scanscope XT into that of the images scanned by the Hamamatsu Nanozoomer 2.0-HT by using the stain transfer methods listed in Table 2. The images obtained using the aforementioned scanners and the ground truth were then used to calculate the SSIM and PSNR, and the corresponding results are presented in Table 2. The results presented in Table 2 indicate that the GAN-based methods achieved higher mean SSIM and mean PSNR values than did the conventional image processing methods. This result indicates that GAN models are crucial for biopsy stain transfer tasks. In addition, S3CGAN achieved higher mean SSIM and mean PSNR values than did the other methods. To understand whether the method proposed in this study achieves statistical significance over other methods on the SSIM and PSNR, *p*-values were also calculated. The results show that all reach statistical significance. Thus, the model developed in this study has excellent fidelity capability for histological image stain transfer, which is crucial for histological image interpretation. Information on histological image structures, such as the shapes, distributions, and the relative positions of the cell nucleus and cytoplasm, can affect the clinical interpretation outcome.

### 4.4. Specialized Color Classifier vs. Embedded Classifier

**Figure 11:**
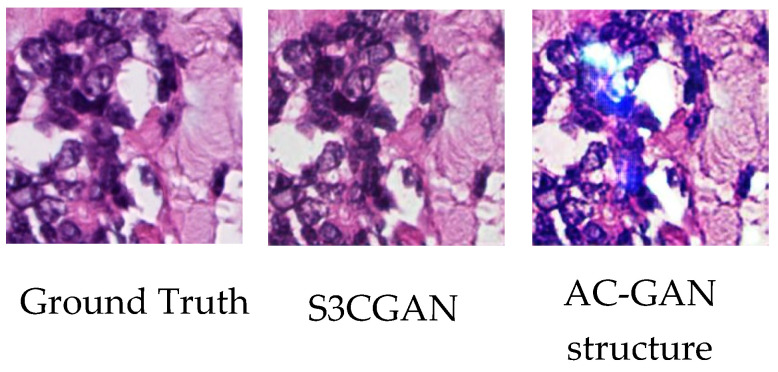
Comparison of the results obtained using the specialized color classifier and embedded classifier.

This section presents detailed descriptions of the specialized color classifier and embedded classifier. The specialized color classifier used by the S3CGAN is a simple and pretrained convolutional neural network architecture. Therefore, it can support GAN training in the initial training phase and assist in optimizing the generator performance. The embedded classifier is a branch network of the discriminator and is still learning during the initial training phase; thus, it cannot effectively assist in optimizing the generator performance during the training phase. The abnormal color spots in Figure 11 appear when the embedded classifier is used in the training process.

### 4.5. Training Stability and the Hyperparameters of the Specialized Color Classifier

**Figure 12:**
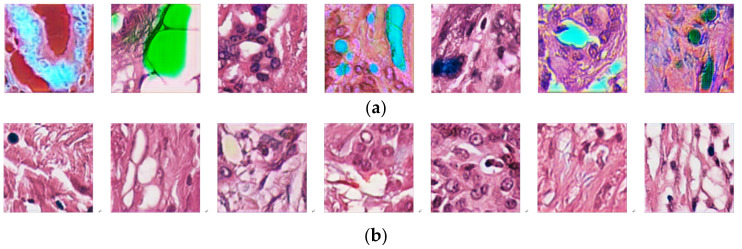
Seven randomly selected snapshots from network outcomes of seven independent runs, (**a**) without and (**b**) with the specialized color classifier using S3CGAN architecture.

To understand the effect of the specialized color classifier on the training stability of the stain style conversion, we removed the specialized color classifier from S3CGAN and conducted seven independent runs. In each run, the network was initialized first and then took a snapshot for a randomly selected outcome after 1000 epochs. The seven snapshots are shown in Figure 12a. Using the same process, seven snapshots of S3CGAN’s outcomes were also taken, and the results are shown in Figure 12b. From Figure 12, we can find that adding the specialized color classifier can indeed make the color of the image generated by the network more accurate in the iterative process.

In this study, we used two hyperparameters in Equation (5), namely α and β. The parameter α is the weight for the cycle consistency and the parameter β is the weight for the color classification loss. We followed the parameter setup of the CycleGAN and set α as 10. Table 3 presents the different values adopted for β. We found that when β was between 0.1 and 1, the generated stain color transfer results did not differ considerably; however, the overall color contrast of the generated image was stronger when β was set as 1. A stronger color contrast is more suitable for tumor classification. Hence, we set the β value as 1 when performing stain transfer.

## 5. Conclusions

This study proposes a new stain transfer model based on the CycleGAN called S3CGAN. This model uses the end-to-end characteristic of neural network architectures to overcome the need of selecting reference images manually. The cyclic design of the CycleGAN enables it to overcome the limitation of the CGAN, which requires paired inputs. To improve the fidelity of the generated image, the S3CGAN model utilizes a U-Net-based generator with excellent structural detail expression ability and a Markovian-based discriminator with local receptive fields. For maintaining training stability, the proposed model uses the WGAN–GP during training to prevent unbalanced training due to the performance difference between the generator and the discriminator. In addition, we propose the use of a specialized color classifier as a “color consultant”, which can increase the stability of network training and improve the quality of the generated image. The experimental results indicate that, compared with conventional image processing methods and GAN-based methods, the proposed S3CGAN generates images with superior color and textural structures. The S3CGAN has excellent fidelity capability for histological mage stain transfer, which is a crucial capability for histological image interpretation.

## Figures and Tables

**Table 1:** AUC values obtained with each stain transfer method after the transfer outcome was input into the tumor classifier.

Methods	AUC (Simple Classifier)	AUC (Complicated Classifier)
Reinhard [1]	0.58	0.83
Macenko [2]	0.65	0.85
Vahadane [3]	0.77	0.87
Cho [7]	0.73	0.90
StainGAN [9]	0.79	0.91
AC-GAN structure	0.81	0.91
S3CGAN*	0.81	0.91
S3CGAN	0.83	0.92

**Table 2:** Mean SSIM and mean PSNR values for different stain transfer methods.

	SSIM/*p*-Value	PSNR/*p*-Value
Reinhard [1]	0.58/0.00	13.4/0.00
Macenko [2]	0.67/0.00	14.0/0.00
Vahadane [3]	0.65/0.00	14.2/0.00
Cho [7]	0.68/0.00	20.4/0.00
StainGAN [9]	0.73/0.00	23.0/0.00
AC-GAN structure	0.69/0.00	21.1/0.00
S3CGAN*	0.75	24.7
S3CGAN	0.76	24.9

**Table 3:** Stain Transfer Results Obtained with the S3CGAN When Using Different β Values.

	Source	β = 0.1	β = 0.3	β = 0.4	β = 1
Stain A to B	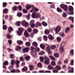	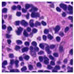	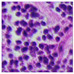	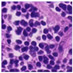	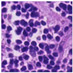
Stain A to B	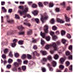	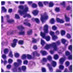	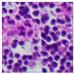	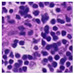	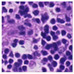
Stain B to A	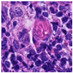	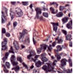	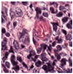	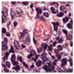	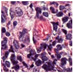
Stain B to A	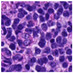	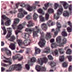	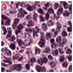	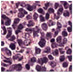	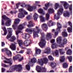

## Data Availability

Not applicable.
